# Preparation of Symmetrical Capacitors from Lignin-Derived Phenol and PANI Composites with Good Electrical Conductivity

**DOI:** 10.3390/ijms24108661

**Published:** 2023-05-12

**Authors:** Penghui Li, Jiangdong Yu, Mingkang Wang, Wanting Su, Chi Yang, Bo Jiang, Wenjuan Wu

**Affiliations:** 1 Jiangsu Co-Innovation Center of Efficient Processing and Utilization of Forest Resources, Nanjing Forestry University, Nanjing 210037, China; liph@njfu.edu.cn (P.L.);; 2 College of Light Industry and Food Engineering, Nanjing Forestry University, Nanjing 210037, China; jiangdongyu@njfu.edu.cn (J.Y.); wmk@njfu.edu.cn (M.W.);

**Keywords:** lignin, additives, polyaniline, conductive polymers, supercapacitors

## Abstract

As a natural polymer, lignin is only less abundant in nature than cellulose. It has the form of an aromatic macromolecule, with benzene propane monomers connected by molecular bonds such as C-C and C-O-C. One method to accomplish high-value lignin conversion is degradation. The use of deep eutectic solvents (DESs) to degrade lignin is a simple, efficient and environmentally friendly degradation method. After degradation, the lignin is broken due to β-O-4 to produce phenolic aromatic monomers. In this work, lignin degradation products were evaluated as additives for the preparation of polyaniline conductive polymers, which not only avoids solvent waste but also achieves a high-value use of lignin. The morphological and structural characteristics of the LDP/PANI composites were investigated using ^1^H NMR, Fourier-transform infrared spectroscopy, scanning electron microscopy, transmission electron microscopy, thermogravimetric analysis and elemental analysis. The LDP/PANI nanocomposite provides a specific capacitance of 416.6 F/g at 1 A/g and can be used as a lignin-based supercapacitor with good conductivity. Assembled as a symmetrical supercapacitor device, it provides an energy density of 57.86 Wh/kg, an excellent power density of 952.43 W/kg and, better still, a sustained cycling stability. Thus, the combination of polyaniline and lignin degradate, which is environmentally friendly, amplifies the capacitive function on the basis of polyaniline.

## 1. Introduction

With the development of a low-carbon economy, the demand for biomass around the world is increasing. Lignocellulosic biomass has broad prospects for development as a sustainable material to reduce fossil resource use [1]. Unlike cellulose and hemicellulose, lignin is often disposed of as waste or fuel, and only 5–10% of lignin is used for high-quality and high-value production. As the most widely sourced renewable aromatic biomass, lignin is the most promising renewable raw material for the preparation of alternative petrochemicals [2]. The three main precursors of the lignin macromolecule are *p*-coumaryl alcohol, coniferyl alcohol and mustard alcohol. Different sources of lignin result in different levels of these precursors. Lignin from softwoods contains mainly *p*-coumaryl alcohol at about 90–95%, lignin from hardwoods usually contains coniferyl alcohol and mustard alcohol at about 25–50% and 50–75%, respectively, while lignin from grasses usually contains all three monomeric alcohols. Lignin polymers have carbon–carbon and ether bonds, the major bond being the ether bond, which accounts for 56% or more of the total bond. Upon depolymerization, lignin can yield a variety of fuels and low-molecular-weight chemicals, including phenol, which can alleviate the overuse of petroleum fuel resources [3]. In recent years, deep eutectic solvents (DESs) have been successfully used in the depolymerization and modification of lignin. Compared with other degradation methods, DES has mild reaction conditions and can achieve high-efficiency degradation of lignin. The degraded lignin has a high hydroxyl content [4].

A common solid electrode material in supercapacitors is polyaniline (PANI), a typical *p*-type conducting polymer [5] because it is simple to manufacture, has high pseudocapacitance (>800 F/g) and excellent multiplicative performance [6]. However, excessive oxidation and repeated volume changes during long charge/discharge cycles can lead to a gradual performance degradation [7]. As an electrochemically active additive, polyaniline needs to function in acidic media, requiring the resulting protonated polyaniline (emerald salt) to have high solubility properties in electrolyte solvents. In addition, the high theoretical capacity of PANI is limited by poor PANI usage due to PANI’s electrical conductivity and the doped ions’ transport channel [8]. Kikuchi et al. [9] synthesized polyaniline/lignin composites in various ratios through oxidative polymerization reactions, and the addition of lignin led to improved thermal resistance of PANI. Conductive polymer dopant substances can sufficiently alter the electronic, magnetic or structural properties of the polymer, and the electrical conductivity can be increased substantially. Secondary doping with lignin may turn dense coils into linearly expanded coils and increase conductivity [10]. In order to reduce the electrochemical flaws of PANI as an electrode material, numerous PANI-based composites have been designed and prepared. When redox-active biopolymers (e.g., lignin) are combined with conductive polymers, improvements in charge storage quality can be achieved [11]. As lignin is electrically insulating, it is the lignin and the conductive substrate that enable the storage and transfer of electrons [12]. The quinone structure of lignin and its derivatives will exhibit strong redox activity and contribute to charge-transfer reactions between electrode surfaces and soluble substances [13]. Industrial lignin is relatively common, and it is chemically and structurally heterogeneous, which is due to its plant-based origin and extraction methods. Therefore, the development of an effective method for lignin separation and degradation could address issues related to lignin heterogeneity and molecular weight distribution [14]. Lignin-derived pseudocapacitive materials are subject to rapid self-discharge, and high self-discharge rates can lead to a loss of energy and power density [12,15]. In order to control the cost of supercapacitors, it is crucial to design an inexpensive hybrid electrode of conductive materials interacting synergistically with lignin.

Gonçalves et al. [16] used pristine cardanol as the main additive to synthesize conductive polyaniline; the resulting material exhibited semiconducting behavior (achieving a conductivity of 9.7 × 10^−1^ S/cm), and some nanofibers were present in the resulting morphology. Aoyagi et al. [17] prepared lignin phenol products from lignocellulose, and the phenolized compounds were rich in phenolic hydroxyl groups, thereby demonstrating better electrical conductivity. It is worth mentioning that the lignin phenol conductivity of *Tsuga heterophylla* was 4.6 × 10^−6^ S/cm. Compounding it with PANI through polymeric structures, hydrophobic π–π stacking and intermolecular interactions produced excellent electrical conductivity (emerald-green imine conductivity of 1–5 S/cm). Inspired by this, the present work prepared highly cross-linked rod-like lignin degradation product/polyaniline (LDP/PANI) composites through the chemical oxidative polymerization of alkaline lignin DES degradates with aniline and investigated the electrochemical properties of the composites by cyclic voltammetry, galvanostatic charge/discharge tests and electrochemical impedance spectroscopy. It is worth mentioning that we have previously explored the capacitive properties of lignosulfonate with polyaniline composites (through the reaction of the sulfonic acid groups in them with polyaniline) [18]. There are many types of lignin, and alkaline lignin’s are also relatively common and inexpensive. This paper provides a quick and highly productive alkaline lignin treatment that allows alkaline lignin to be well compounded with polyaniline as well, broadening the range of lignin species that can be compounded with polyaniline. The incorporation of LDP mitigated to some extent the structural collapse of PANI induced during multiple charge–discharge processes. The capacity retention of the LDP/PANI was also much higher than that of PANI. Thus, it can be seen that the addition of LDP has a significant benefit on its stability.

## 2. Results and Discussion

**Figure 1:**
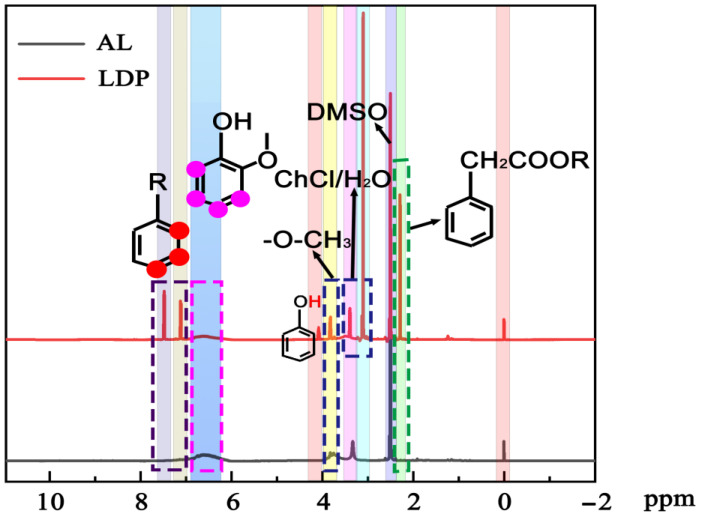
^1^H NMR spectra of AL and LDP obtained by using a 600 MHz spectrometer (24 mg/mL of DMSO-*d*6).

Figure 1 shows the ^1^H NMR spectra before and after degradation by DES. The two peaks are located at 7.5–7.0 ppm, which are assigned to the ortho-, para- and meta- positions on the benzene ring, respectively. The broad peak at 7.0–6.3 ppm is attributed to the aromatic proton in the guaiacyl unit [19], 4.0–3.5 ppm to the methoxy proton signal, the sharp peak at 3.5–3.3 ppm to the proton signal of water in the solvent and the small peak at 3.4 ppm to the proton signal in choline chloride. During treatment, there may have been some interaction between the lignin and the solvent choline chloride, which may have caused this peak to form, or there may have been a small quantity of deep eutectic solvent left over [4]. The sharp peak at 2.5 ppm was attributed to the proton signal of undeuterated dimethyl sulfoxide in deuterated dimethyl sulfoxide. The peak at 2.5–2.2 ppm was caused by the proton signal of the aromatic ring acetate, and it can be shown that, after degradation, the strength of the acetate signal of lignin greatly increased. In addition, the 4.3–4.0 ppm peak was caused by the phenolic hydroxyl proton signal, and it can be seen that a stronger phenolic hydroxyl proton signal appeared after degradation, presumably resulting from the breakage of the β-*O*-4 bond.

**Figure 2:**
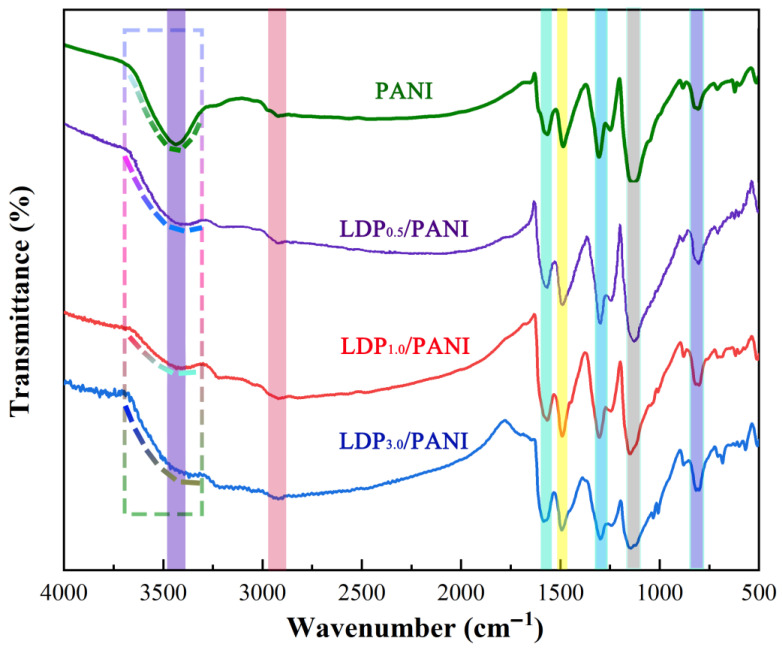
FTIR spectra of lignin derivatives and PANI composite samples.

Figure 2 shows the FTIR spectra of PANI with the lignin derivative/PANI; it can be seen that the complex also exhibits the characteristic peaks of PANI. The peak at 1563 cm^−1^ is owing to the quinone structure of PANI. The other strong signal at 1487 cm^−1^ is due to the benzene-type structure of PANI. The peaks at 1301 cm^−1^ and 1113 cm^−1^ are assigned to the CN deformation of the benzene ring unit and the CH bending vibration of the N=Q=N segment, respectively [16]. We were also surprised to find that the absorption of the N-H stretching pattern was observed at around 3500 cm^−1^, presumably in the free imine structure. This change in stretching is due to hydrogen bonding between LDP and PANI [17]. The phenolic oligomers degraded in lignin are likely to be involved in the main chain structure of polyaniline by polymerization; the molecular weight of the formed polymer is shown in Table S1. The intensity of the peak at 1728 cm^−1^ increased significantly with the increasing percentage of LDP in the composite. In addition, the strength of the aromatic ring deformation peak at 528 cm^−1^ significantly decreased with the addition of LDP. This shows that the polymer structure’s aromatic rings had become more stable [20].

**Figure 3:**
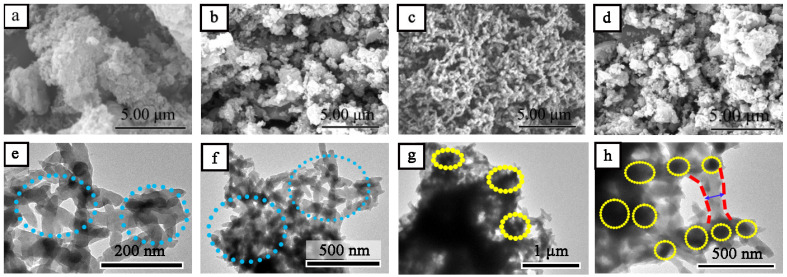
SEM images of (**a**) PANI, (**b**) LDP_0.5_/PANI, (**c**) LDP_1.0_/PANI and (**d**) LDP_3.0_/PANI. TEM images of (**e**,**f**) PANI (the blue circular dotted line marks the distinctive features of the material) and (**g**,**h**) LDP_3.0_/PANI (the yellow circular dashed lines mark the features of the LDP and the red parallel lines mark the diameters of the rod-like structures).

The surface morphology of PANI and the composite is shown in Figure 3. The rod-like structure of the PANI and the LDP particles bonded to the PANI is easily discernible. As the LDP content of the composite rises, a noticeable shift in surface morphology is seen. When LDP is not present, the particulate nanorods are thicker and smoother. The composites show a mixture of nanorods and particles, with the LDP giving the morphology a stable cylindrical shape. The nanorod morphology has a high surface contact area and is the preferred factor in the preparation of conductive polymer composites. Figure 3 depicts PANI and LDP nanocomposites forming tightly bound nanocomposites rooted in a polymer matrix [21]. As can be seen in Figure 3e,f, the PANI nanorods are well defined and have a diameter of around 120 nm (spacing of red lines as in Figure 3h). In Figure 3g,h, we can see that the LDP/PANI composite is more likely to cross, and the addition of LDP “wraps” each nanorod, making each nanorod thicker. A closer look reveals that the radius of each nanorod is around 40 nm. Based on previous reports, it is assumed that this interwoven nanorod structure must be largely due to the heterogeneous nucleation of aniline polymerization [22]. In addition, the LDP/PANI composite is a “three-dimensional interweave”, a composite structure that solves the nanorod breakage of pure PANI during electrode charging and discharging and acts more like a protective layer. This structure also provides a structural basis for the transfer and storage of electrons [23].

**Figure 4:**
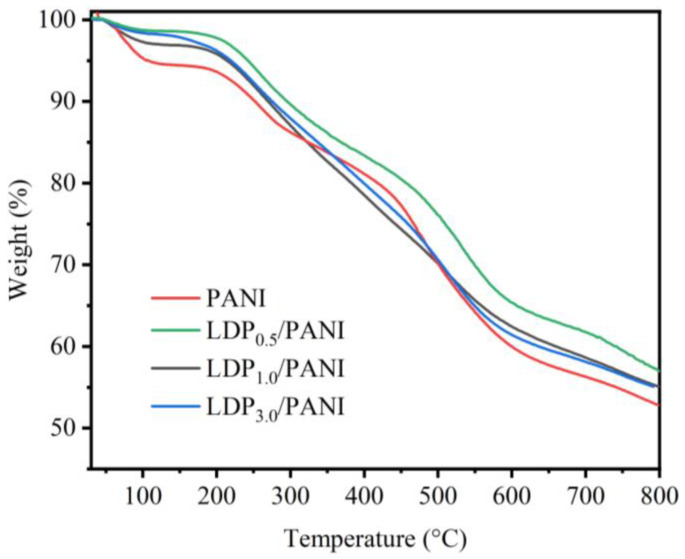
TGA curves of PANI, LDP_0.5_/PANI, LDP_1.0_/PANI and LDP_3.0_/PANI.

The TGA curves for the PANI, LDP_0.5_/PANI, LDP_1.0_/PANI and LDP_3.0_/PANI composites are shown in Figure 4. During the initial heating stage, the temperature was raised from 30 °C to 110 °C, at which point, the water molecules detached from the samples, and a small weight loss occurred in all four samples. The thermal degradation phase of the four curves for the PANI, LDP_0.5_/PANI, LDP_1.0_/PANI and LDP_3.0_/PANI composites then started at around 190 °C, and at about 300 °C, the rate was the fastest, with PANI losing weight at the fastest rate. Comparing the LDP/PANI curves with the PANI curves, the LDP/PANI composite’s curves were consistently higher than the PANI ones, suggesting that the thermal stability of the LDP/PANI composites was superior to that of the PANI. This may be due to the reaction between the rigidity of the lignin chains and the PANI single chain, with some of the aniline monomer acting as a diffusion chain for the LDP/PANI composites [24]. A slight weight loss was also found at around 500 °C and 750 °C. This could be due to oxidative decomposition. Furthermore, it is readily apparent that LDP_0.5_ and PANI may form stronger bonds than LDP_1.0_- and LDP_3.0_-doped PANI, presumably due to hydrogen bonding [25].

**Figure 5:**
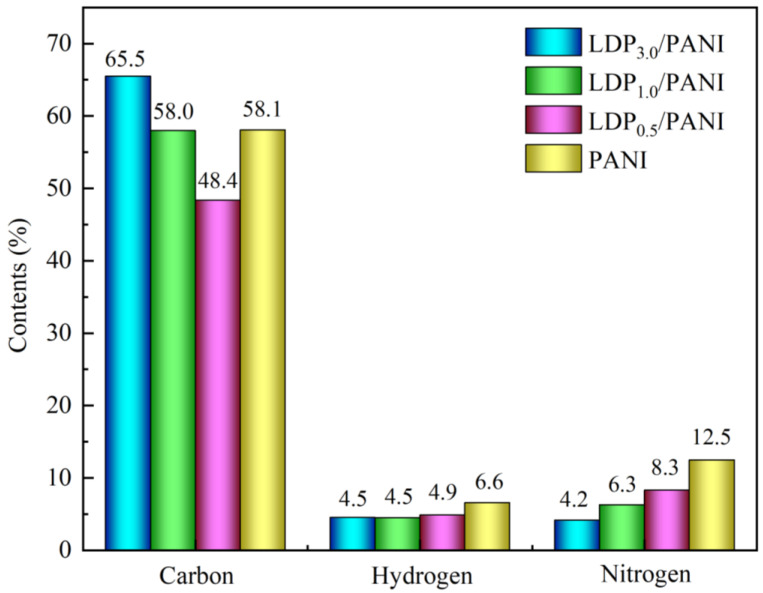
Elemental analysis distribution diagram of PANI, LDP_0.5_/PANI, LDP_1.0_/PANI and LDP_3.0_/PANI.

Figure 5 shows the distribution of C, H and N elements analyzed for PANI, LDP_0.5_/PANI, LDP_1.0_/PANI and LDP_3.0_/PANI. We can find that the C, H and N contents of PANI and doped LDP are essentially the same, but the addition of LDP makes the C/N ratio of the LDP/PANI composite somewhat larger. This is also in line with the different doping of LDP, which itself has a higher carbon content, and the different doping gives a gradient in the carbon content of the composite [18].

**Figure 6:**
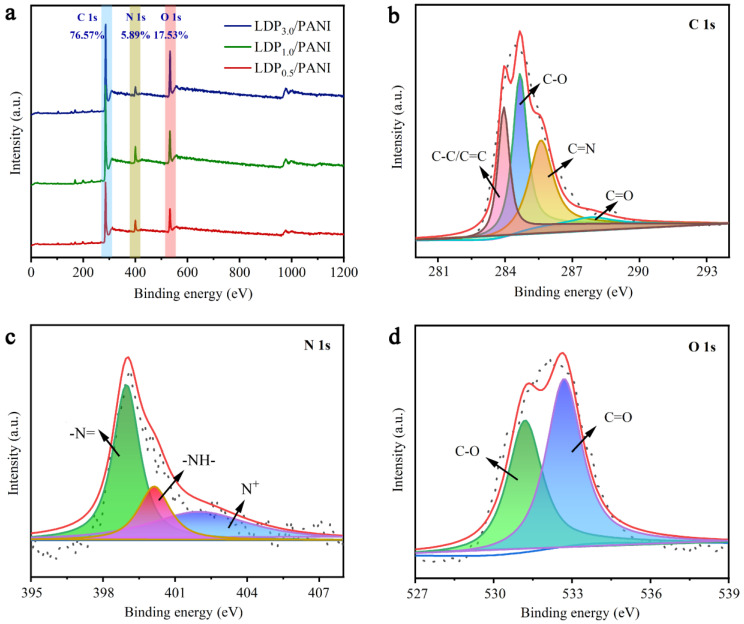
(**a**) High-resolution XPS spectra of LDP_0.5_/PANI, LDP_1.0_/PANI and LDP_3.0_/PANI; (**b**) C 1s XPS spectra of LDP_3.0_/PANI; (**c**) N 1s XPS spectra of LDP_3.0_/PANI; (**d**) O 1s XPS spectra of LDP_3.0_/PANI.

Figure 6a shows the full XPS spectra of LDP_0.5_/PANI, LDP_1.0_/PANI and LDP_3.0_/PANI. It can be seen that the LDP/PANI composites contain mainly three elements, i.e., C, N and O. The LDP_3.0_/PANI contains 76.57% C, 5.89% N and 17.53% O, which is more similar to the elemental analysis. The C 1s spectra of the LDP_3.0_/PANI composites show peaks at 283.3 eV, 284.4 eV, 285.1 eV and 288.4 eV corresponding to the aromatic C-C/C=C, C-O, C-N and C=O/O-C=O, respectively (as shown in Figure 6b). As can be observed from Figure 6c, the N 1s spectrum of the LDP_3.0_/PANI composite is 398.9 eV for the undoped imine structure (-N=), 399.9 eV for the undoped amine structure (-NH-) and 402.5 eV for the protonated amine structure (N^+^). The presence of -N= and -NH- in the above peaks also reflects the fact that supercapacitors can have pseudocapacitance [26]. As seen in Figure 6d, the C=O and C-O in the O 1s spectrum appear at 532.7 eV and 531.3 eV. The above XPS spectra indicate that the aniline oxidation polymerization successfully doped the LDP. The non-protonated amine fraction is very close to the protonated amine and imine nitrogen fractions, and the LDP_3.0_/PANI composite is in the emerald oxide state with a more positively charged nitrogen fraction. The LDP steric structure in the PANI matrix will have more active sites [27]. In addition, the higher N^+^ amine content of the complexes and the relatively high N content is a result of the interaction between the lignin hydroxyl group and the PANI amine group through the formation of hydrogen bonds [28].

The electrical conductivity of polyaniline depends mainly on the doping rate and the degree of oxidation. The molecular structure of polyaniline is doped with oxidation and reduction units (see Figure S1), which reversibly changes the conductivity of polyaniline from the insulating form (undoped) to the conducting form (doped), with a conductive emerald-green imine conductivity of 1–5 S/cm. Table 1 displays the conductivity (σ) variation with varying amounts of LDP added to PANI as measured by the standard four probe method at room temperature. The conductivity of LDP/PANI compared to PANI ranged from 0.57 S/cm to 1.36 S/cm. The conductivity continued to increase further but at a slower rate as the amount of LDP doped in PANI increased (Table 1). LDP acts as the main additive in the polymerization process, which maximizes the number of carriers [29]. LDP acts as an additive to produce polymers with the properties of semiconductor materials [16].

**Figure 7:**
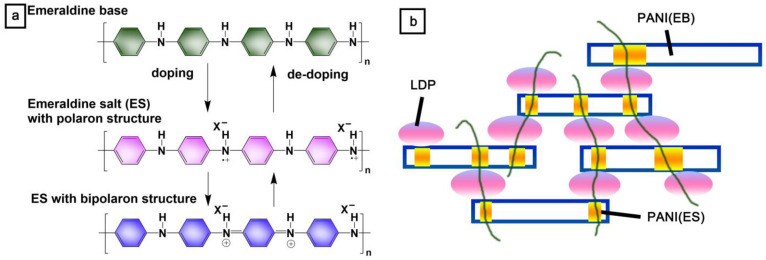
(**a**) Various structures of polyaniline groups; (**b**) schematic model of electrical conductivity of LDP/PANI composites, adapted with permission from Ref. [17].

Regarding the reasons why LDP incorporation affects the conductivity, i.e., the mechanisms of organic (semi)conductor doping, there should be two aspects: Firstly, LDP is a small and homogeneous polymer (M_w_ = 3.6 × 10^3^ g/mol, PDI = 1.8, Table S1) and the breaking of the ether bond leads to an increase in phenolic hydroxyl groups, promoting the doping of PANI. It can be observed in Figure 2 that there is less carboxylic acid in LDP, indicating that most of the phenolic hydroxyl groups are involved in the acidic protonated doping. Secondly, the increased interaction between LDP and PANI also promotes electron mobility (Figure 7). It has been suggested that the doping of LDP may be due to the π–π stacking of phenolic hydroxyl groups and aromatic rings [17].

To further investigate the feasibility of PANI and LDP/PANI composites as electrode materials for supercapacitors, a symmetrical, liquid-double-electrode system made of PANI or LDP/PANI composites was tested using the CV and GCD methods. Two equal-mass electrode sheets of PANI or LDP/PANI composite material were assembled into a push-button symmetrical supercapacitor in the order of cell case, positive electrode, spacer, negative electrode and cell case [30]. Electrochemical measurements were carried out in a 1 M H_2_SO_4_ solution with a potential window of 0 to 1 V. Afterward, the following results were obtained from tests on the two-electrode system.

**Figure 8:**
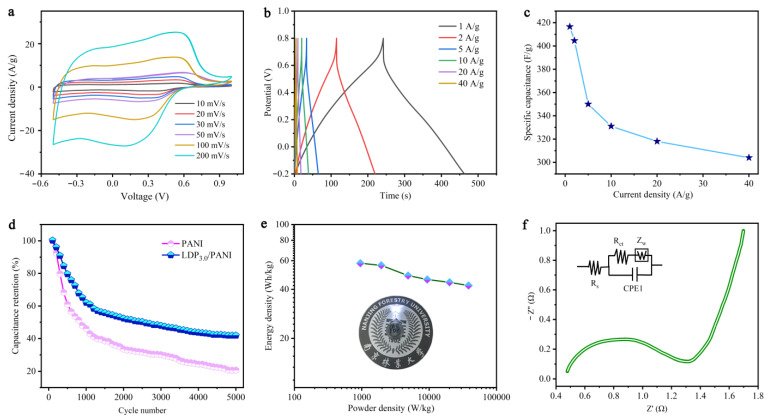
(**a**) CV curves of LDP_3.0_/PANI obtained at the scan rate of 10–200 mV/s; (**b**) charge–discharge curves of LDP_3.0_/PANI obtained at a current density of 1–40 A/g; (**c**) specific capacitance retention of LDP_3.0_/PANI with varied current densities from 1 to 40 A/g; (**d**) cycling stability of PANI and LDP_3.0_/PANI at 5 A/g for 5000 cycles; (**e**) ragone plot of the LDP_3.0_/PANI symmetric supercapacitor (the illustration shows a photo of a small light bulb being lit by an assembled supercapacitor); (**f**) electrochemical impedance spectra of LDP_3.0_/PANI at open-circuit voltage (illustration shows an analog circuit diagram, the green line is the fitted EIS curve).

The presence of PANI, which inevitably leads to pseudocapacitance, is also evidenced by the CV curve results [31]. As can be seen in Figure 8a, the CV curves for the LDP_3.0_/PANI composites were measured at scan rates of 10–200 mV/s over a potential window of −0.5 to 1.0 V. There is a redox peak at around 0.6 V supposedly caused by the intermediate of hydroquinone/benzoquinone [32]. The CV curve measured at a scan rate of 200 mV/s reveals a more prominent redox peak and exhibits the desired capacitive behavior. As the scan rate rises, the CV curve area tends to increase and the CV curve shape changes regularly, with the reduction peak (trending toward negative) and the oxidation peak moving in opposite directions. High scan rates result in shorter electrolyte ion diffusion times, inadequate redox reactions and a small rise in the resistance of the electrode. To better investigate the electrode performance, charge and discharge measurements were carried out at a current density of 1–40 A/g, from −0.2 to 0.8 V, as shown in Figure 8b. The discharge curves of LDP_3.0_/PANI composites exhibit two different voltage levels, the first from 0.8 to 0.6 V and the second from 0.6 to −0.2 V, respectively [33,34]. The bilayer capacitance is the reason for the relatively short discharge duration in the first stage, while the combined effects of the bilayer and Faraday capacitances account for the longer discharge duration in the second stage. According to the charge and discharge curves in Figure 8b, the specific capacitance of LDP_3.0_/PANI composites at 1 A/g is 416.6 F/g, which corresponds to the CV curve. As demonstrated in Figure 8c, the specific capacitance of LDP_3.0_/PANI composites is approximately 304.5 F/g when the current density is at 40 A/g, indicating a capacity retention of 72.9% (compared to 1 A/g); a result that in a capacitive performance is equal to or even more stable than that of most PANI-based composites (Table 2). In order to evaluate the cycling performance of the material, cycling stability was evaluated at a current density of 5 A/g with a cycle count of 5000, and it can be found that, after 5000 cycles, LDP_3.0_/PANI composites reached 41.88% compared to 20.67% for PANI (Figure 8d). This is due to the potential for the deprotonation and volume expansion/contraction of PANI over long cycles. Another major indicator regarding the button double-electrode system is the energy and power density. As seen in Figure 8e, our electrodes show an energy density of 57.86 Wh/kg and a power density of 952.43 W/kg, reflecting the practicality and feasibility of the electrodes [31]. Furthermore, to assess the kinetic properties of the electrochemical processes in the LDP_3.0_/PANI composites, it is necessary to observe the EIS Nyquist diagram, and Figure 8f shows the data and the results of the fitting. It can be observed that the curve shape of the LDP_3.0_/PANI composites in the high-frequency region resembles a semicircle, exhibiting a combined behavior of double-layer capacitance and charge-transfer resistance (R_ct_). The intercept of the curve on the *x*-axis reflects the internal resistance of the whole system, while the diameter of the semicircle of the curve represents the electrochemical response at the electrolyte–electrode interface. LDP_3.0_/PANI composites show a small semicircle diameter (R_ct_ 0.9 Ω, smaller compared to PANI) [18], probably because of the more dispersed nanorods in the structure and the high electrical conductivity. The high slope indicates that it has a faster capacitive response, which is due to the transfer in the ion interpenetrating framework structure [35].

## 3. Materials and Methods

### 3.1. Raw Materials and Reagents

Alkaline lignin (AL, M_w_ = 6483) was purchased from Aladdin Reagent Co. From Nanjing Maclean’s Reagent Company, conductive carbon black, polyvinylidene fluoride (PVDF), aniline (when used, twice distilled under reduced pressure), choline chloride, *p*-toluenesulfonic acid and ammonium persulfate were purchased. Concentrated hydrochloric acid was of domestic analytical purity, and the lab water was ultrapure water.

### 3.2. Preparation of Lignin Degradation Product/Polyaniline (LDP/PANI) Composites

Alkaline lignin degradation was carried out as stated in the literature [4]. At 130 °C, *p*-toluenesulfonic acid and choline chloride were mixed in a 1:1 molar ratio for about 30 min to form a colorless and clear solution, which was stored in a desiccator (the bottom of the desiccator held the desiccant, which was a color-changing silica gel, at room temperature). To the reaction flask, 1 g of lignin and 19 g of deep eutectic solvent were added to give a bulk fraction of 5% lignin. Next, 250 µL of water that was distilled and heated at 130 °C for 5 h was added. At the end of the reaction, the reaction flask was removed and quickly immersed in a cold-water bath to end the reaction. Subsequently, 50 mL of acidic water (HCl, pH = 2) was added to the reaction flask to precipitate the lignin, centrifuged to give a solid and then freeze-dried to give the degradation product, labeled lignin degradation product (LDP).

**Scheme 1:**
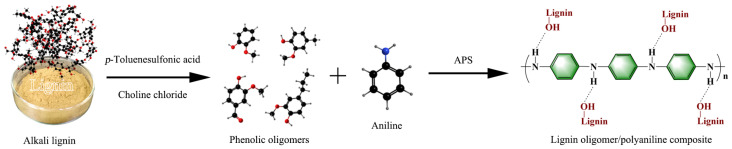
Synthesis mechanism diagram of LDP/PANI.

Based on our previous work, the lignin degradation product/polyaniline composite was prepared by chemical oxidative polymerization [18], as shown in Scheme 1. A 50 mL solution of 1.0 M HCl was used to disperse 1.0 g of LDP. The aforementioned suspension received 0.91 mL of aniline, which was then stirred magnetically at 0 °C for two hours. In addition, 2.30 g of ammonium persulfate (APS) was quantified and dissolved in 50 mL of a 1.0 M HCl solution as an oxidant. The aforementioned solution was blended in a 1:1 molar ratio of aniline to APS and oxidized chemically in situ at 0 °C for 24 h to polymerize. The reaction product was filtered, and the filtrate underwent a series of washings with distilled water until it reached a pH of 7 and was dried under vacuum to a constant mass to obtain the LDP_1.0_/PANI composite. The composites were produced by adding 0 g, 0.5 g and 3 g of LDP using the aforementioned procedure and were recorded as PANI, LDP_0.5_/PANI and LDP_3.0_/PANI, respectively.

The detailed characterization and the test methods for the electrochemical performance of the electrodes can be found in the Supporting Information.

## 4. Conclusions

In this work, the lignin degradation products were prepared in one step using DES and doped in PANI to achieve a high-value application of lignin; the prepared composites exhibited a semiconducting behavior (achieving a conductivity of 1.36 S/cm). The morphology of the obtained composites contains many nanorods that contribute to the enhancement of the electrical conductivity. These results indicate that LDP is a promising additive. Due to the addition of LDP, the rod-like structure of the compound is protected by a “protective film” and retains its three-dimensional network structure. The best LDP/PANI composite as an electrode material for supercapacitors has a large specific capacitance of 304.5 F/g at a large current density of 40 A/g. As the current density increased from 1 A/g to 40 A/g, the LDP/PANI composite with a higher proportion of LDP remained at 72.9% of the initial specific capacitance. Furthermore, at a current density of 5.0 A/g, the LDP/PANI assembled as a supercapacitor showed a capacity retention of 41.88% after 5000 cycles, far exceeding the 20.67% of PANI. This work provides a direction for value addition to lignin.

## Figures and Tables

**Table 1:** Conductivity of samples before and after doping with lignin derivatives.

Samples	Conductivity (S/cm)
Pure PANI	0.18
LDP_0.5_/PANI	0.57
LDP_1.0_/PANI	1.10
LDP_3.0_/PANI	1.36

**Table 2:** Comparison of the performance of supercapacitors prepared from different polyaniline bases.

Samples	Current Density (A/g)	Specific Capacitance (F/g)	Cycle Numbers	Capacitance Retention (%)	Ref.
Ligninsulfonate/PANI	1	554	5000	55	[18]
3D hollow rGO/PANI ball	1	331	500	86	[36]
PANI nanowire/ NA/CA	0.3	402	1000	61	[37]
Porous PANI film	0.25	361	500	72.3	[38]
Porous PANI-CNTs	0.5	407	2200	55	[39]
GR-PANI hollow sphere	0.5	456	1000	83	[40]
PANI-GO composite	1	442	2000	83	[41]
PANI-C nanotubes	0.5	483	2000	63	[42]
Hollow PANI nanofiber	1	601	500	62	[43]
Hollow PANI/rGO sphere	1	614	500	90	[44]
DMSO-PEDOT:PSS/PANI	1	340	5000	69	[45]
LDP_3.0_/PANI	1	417	5000	42	This work

## Data Availability

Not applicable.

## Supplementary Materials

The following supporting information can be downloaded at: https://www.mdpi.com/article/10.3390/ijms24108661/s1, Figure S1: Five oxidation states of polyaniline and digital photos of LDP_3.0_/PANI; Table S1: Molecular weight of AL, LDP, PANI, and LDP_3.0_/PANI complex.
