# Supplementary material for: Preparation of Symmetrical Capacitors from Lignin-Derived Phenol and PANI Composites with Good Electrical Conductivity
Source: Int J Mol Sci. 2023 May 12;24(10):8661. doi: 10.3390/ijms24108661 (PMC10217818; doi:10.3390/ijms24108661)
Supplement: Supplementary file 1 [file ijms-24-08661-s001.zip › ijms-2322807-supplementary.pdf]

Supporting Information

Material Characterization

^{1}H NMR was acquired by dissolving 12 mg of acetylated lignin sample in 0.5 mL of DMSO-*d6* and shaking to dissolve it. The Bruker AVANCE III 600 MHz NMR instrument with a 5 mm TCl-z gradient triple resonance probe at 25 °C was used for the detection of ^{1}H spectra in the range 0-16 ppm (9615 Hz), with 2048 sampling points in the 1H dimension, a relaxation time of 1.5 s and 64 accumulations. The samples were measured by the KBr compression method on a German VERTEX 80V infrared spectrometer in the wavelength range of 4000-400 cm^{-1}. Environmental scanning electron microscopy (SEM, Quanta 20) with transmission electron microscopy (TEM, JEM-1400) was used to observe the microscopic morphological characteristics of the polyaniline composites before and after LDP doping. The samples were gold sprayed to eliminate static electricity before the SEM test. The samples were prepared by ethanol suspension method before TEM test, dripped on copper mesh, dried and used for observation. An AXIS Ultra DLD X-ray photoelectron spectrometer (XPS) from Shimadzu (Japan), was used to determine the surface chemical composition of the samples, including details of the surface elemental composition and peaks of C (C 1s), O (O 1s), and N (N 1s). The sample detection concentration is greater than 0.1%, the detection depth is less than 10 nm, at least two samples are prepared for each raw material, and at least three different positions are measured for each sample. Include the relative content of elements and the type and relative content of chemical functional groups. The TGA209 F1 thermogravimetric analyzer from NETZSCH, Germany was used to analyze the thermal stability performance of the samples, protected by high purity nitrogen, with an experimental temperature of 30-800°C and a heating rate of 10 °C/min. PerkinElmer EA 2400 II elemental analyzer was used to detect C, H, and N content in the samples. The gel permeation chromatography (GPC) determined the molecular weight and dispersion of lignin samples (LC-20A, Shimadzu, Kyoto, Japan). The concentration of lignin in tetrahydrofuran (THF) was about 5 mg/mL. The column temperature was 40°C, THF was the eluent, and the flow rate was 1 mL/min. The average molecular weight of lignin was measured by an external standard method, in which monodisperse polystyrene was applied as the standard compound.

Electrochemical Measurements

The electronic conductivity of LDP/PANI composite was measured by the conventional four probe technique. Under a pressure of 20 MPa, the sample was pressed into a disk of 2 mm thickness. PANI or LDP/PANI composites were assembled into a symmetrical liquid double electrode system and then subjected to CV and GCD tests. Two equal mass electrode sheets of PANI or LDP/PANI composites material were assembled into a push-button symmetrical supercapacitor in the order of cell case, positive electrode, spacer, negative electrode and cell case. The electrode shell is CR2032, the positive and negative electrode materials are the same material, both are the composite material prepared in this experiment, two carbon papers with similar active material are selected and pressed at 10 MPa, 1 mol/L H_2SO_4 aqueous solution is used as the electrolyte, organic filter membrane is used as the septum, encapsulated in the 2032 electrode shell using the sealing machine, and can be tested electrochemically after 12 h of standing [1].

Electrochemical measurements were carried out in a 1M H_2SO_4 solution with a potential window of 0 to 1V. Afterwards, the following results were obtained from tests

on the two-electrode system. The cyclic voltammetric curves (CV) of the working electrodes were tested in the voltage range of –0.5V to 1V, and different sweep rates (10, 20, 30, 50, 100, 200 mV/s) were used to investigate the multiplicative properties. Galvanostatic charge/discharge curves (GCD) were tested in the voltage range of -0.2 V to 0.8 V. The specific capacitance of the electrode material was calculated according to Equation (1) and obtained by testing conditions at different current densities (1, 2, 5, 10, 20, and 40 A/g). Electrochemical impedance spectroscopy tests were performed on the carbon electrode in the frequency range of 10^{-2}-10^{5} Hz with an amplitude of 5 mV and an initial voltage of the open circuit. The cycle life of the composite electrode was investigated by conducting 5000 cycles of constant current charge/discharge tests at a current density of 5 A/g.

The linear charge/discharge curve equation is shown in (1):

$$C_m = C/m = I\Delta t/(m\Delta v) \quad (1)$$

where C_m (F/g) is the specific capacitance; m (g) is the electrode material loading; I (A) is the charge/discharge current; Δt (s) is the charge/discharge time; ΔV (V) is the voltage window.

The nonlinear charge/discharge curve equation is shown in (2).

$$C_m = \frac{2\times I\times S}{m\times\Delta U^2} = \frac{2\times I\int_{t(U_{max})}^{t(U_{min})} U(t)dt}{m\times\Delta U^2} \quad (2)$$

where: I (A) is the charge/discharge current; S (Vs) is the integrated area under the discharge curve; m (g) is the active material of the electrode; t (Umax) is the time (s) for the start of discharge; t (U_{min}) is the time (s) for the end of discharge; ΔU (V) is the voltage range after deducting the voltage drop.

The energy density and power density are expressed by the following equations (3)-(4):

$$E = \frac{CV^2}{2} \quad (3)$$

$$P = \frac{E}{t} \quad (4)$$

where C (F/g) is the specific capacitance of the supercapacitor, E (Wh/kg) is the energy density of the supercapacitor, V (V) is the operating voltage, P (W/kg) is the power density of the supercapacitor, and t (h) is the discharge time.

Table S1 Molecular weight of AL, LDP, PANI, and $LDP_{3.0}$/PANI complex

	$M_w{}^a$ (g/mol)	$M_n{}^b$ (g/mol)	PDI
AL	7.3×10^3	2.0×10^3	3.6
LDP	3.6×10^3	1.9×10^3	1.8
PANI	75.5×10^3	24.3×10^3	3.1
$LDP_{3.0}$/PANI	68.8×10^3	26.5×10^3	2.6

[a]Weight-average molecular weight; [b]Number-average molecular weight; [c]Polydispersity index (M_w/M_n).

The molecular structure of polyaniline is composed of oxidation units and reduction units. Depending on the degree of reduction, polyaniline is divided into the following states, as shown in Figure S1, including the fully reduced state, the intermediate oxidation state and the fully oxidized state. The all-reduced and all-oxidised states are insulating states, and only the intermediate oxidised state with an equal number of oxidised and reduced units can be turned into a conductor by protonic acid doping. Polyaniline with different degrees of oxidation shows different components, structures, colours and conductive properties, e.g. during the transformation from the fully reduced state to the fully oxidised state, with the increase of oxidation degree polyaniline shows yellow, green, dark blue, dark purple and black.

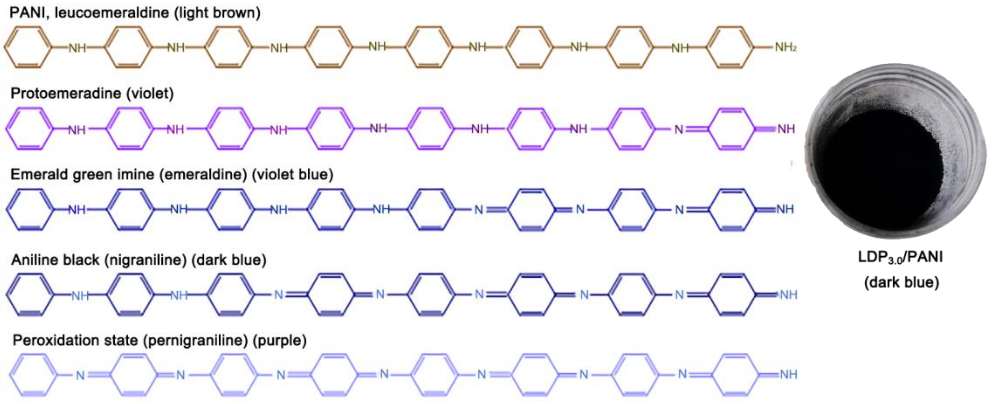

Fig. S1 Five oxidation states of polyaniline and Digital photos of LDP$_{3.0}$/PANI

References

1. Li, P.H.; Wei, Y.M.; Wu, C.W.; Yang, C.; Jiang, B.; Wu, W.J. Lignin-based composites for high-performance supercapacitor electrode materials. *RSC Adv.* **2022**, *12,* 19485-19494.
